# Supplementary material for: An imputation platform to enhance integration of rice genetic resources
Source: Nat Commun. 2018 Aug 29;9:3519. doi: 10.1038/s41467-018-05538-1 (PMC6115364; doi:10.1038/s41467-018-05538-1)
Supplement: Supplementary file 1 — Supplementary Information [file 41467_2018_5538_MOESM1_ESM.pdf]

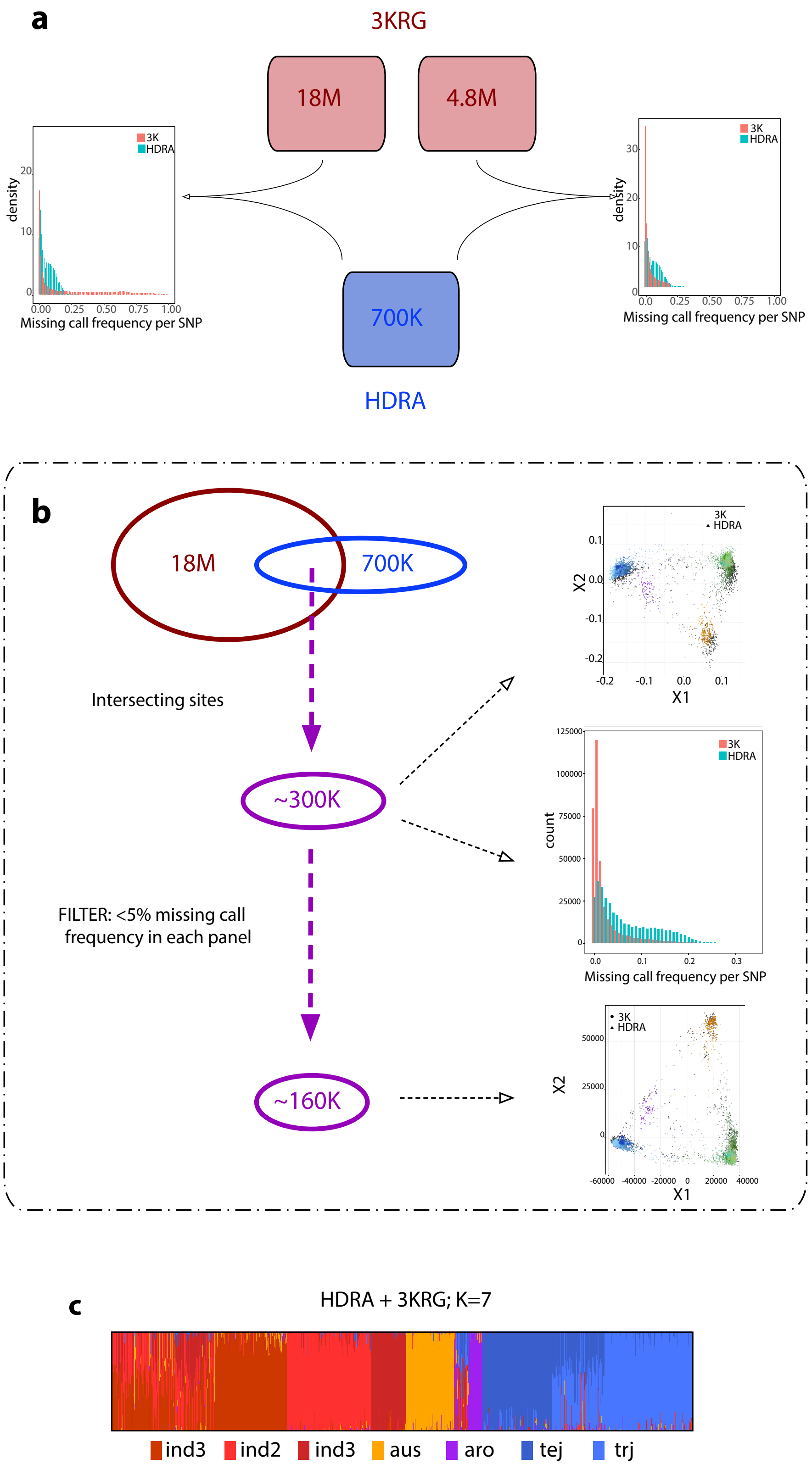

Supplementary Figure 1. Overview of genetic datasets used and produced in this study. **(a)** Distribution of missing data in the HDRA (700K SNPs) and 3KRG (4.8M and 18M SNPs) datasets. **(b)** Using the intersecting sites between the unfiltered 18M 3KG and 700K HDRA datasets, PCA (inset figure, top) of the combined germplasm set (n~4500) displayed an unexpected platform-specific shift (gray samples = HDRA samples; colored = 3KRG samples) due to differential distribution of missing data (inset figure, middle) within the intersecting set of ~300K SNPs. After filtering for missing data in the respective datasets, PCA was free of shift (inset figure, bottom). **(c)** Population structure analysis of the combined HDRA and 3KRG individuals using the ~160K resultant SNPs from (b). The five *O. sativa* subpopulations (*indica, aus, aromatic, temperate, japonica, and tropical japonica*) emerged at K=7, in addition to further stratification of the *indica* subpopulation (ind1, ind2, ind3) due to the large number of *indica* accessions from the 3KRG panel (**Fig. 1**).

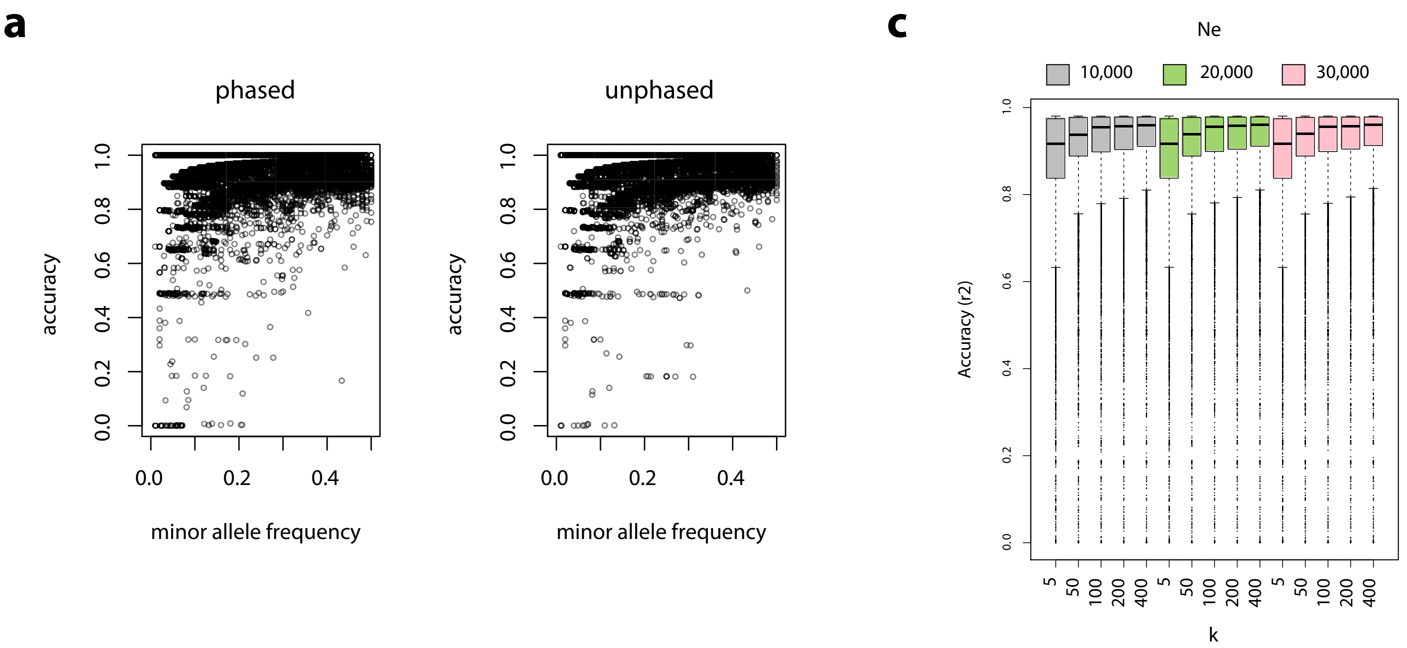

b

khap	Ne	0%	25%	50%	75%	100%
5	10000	4.16E-04	0.978976	1	1	1
5	20000	4.16E-04	0.9790278	1	1	1
5	30000	4.16E-04	0.9790278	1	1	1
50	10000	2.14E-40	1	1	1	1
50	20000	2.14E-40	1	1	1	1
50	30000	2.14E-40	1	1	1	1
100	10000	2.14E-40	1	1	1	1
100	20000	2.14E-40	1	1	1	1
100	30000	2.14E-40	1	1	1	1
200	10000	5.70E-06	1	1	1	1
200	20000	2.14E-40	1	1	1	1
200	30000	2.14E-40	1	1	1	1
400	10000	4.16E-04	1	1	1	1
400	20000	4.16E-04	1	1	1	1
400	30000	4.16E-04	1	1	1	1

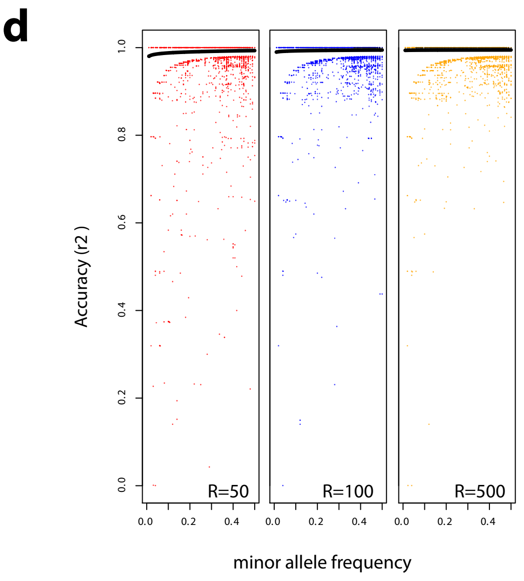

Supplementary Figure 2. IMPUTE2 parameter selection using the Gold Standard panel. (a) Comparison of imputation performance using phased versus un-phased reference panels. No differences were detected between using phased versus un-phased references panels. **(b)** Effect of Ne and k using a full reference panel. Full reference panel contained 2973 diverse re-sequenced samples. Values of 5, 50, 100, 200, and 400 were explored for k while Ne varied from 10,000, to 20,000 and 30,000. For all parameter combinations, median accuracy (computed as site-based squared correlation) were 1.0. For parameter combinations with k > 5, 25% quantiles were also 1.0 (blue shade = r2 of 1.0). Resulting accuracy distributions were nearly equal, so only non-perfect values are displayed in **(c)**. No apparent effect of Ne (gray = 10,000; green = 20,000; pink = 30,000). For k > 5, further increases in k offered slight improvements to accuracy. **(d)** Effect of increasing reference panel size (R= 50, 100, 500) at k = 5. Thick black lines = fitted logarithmic curves of accuracy.

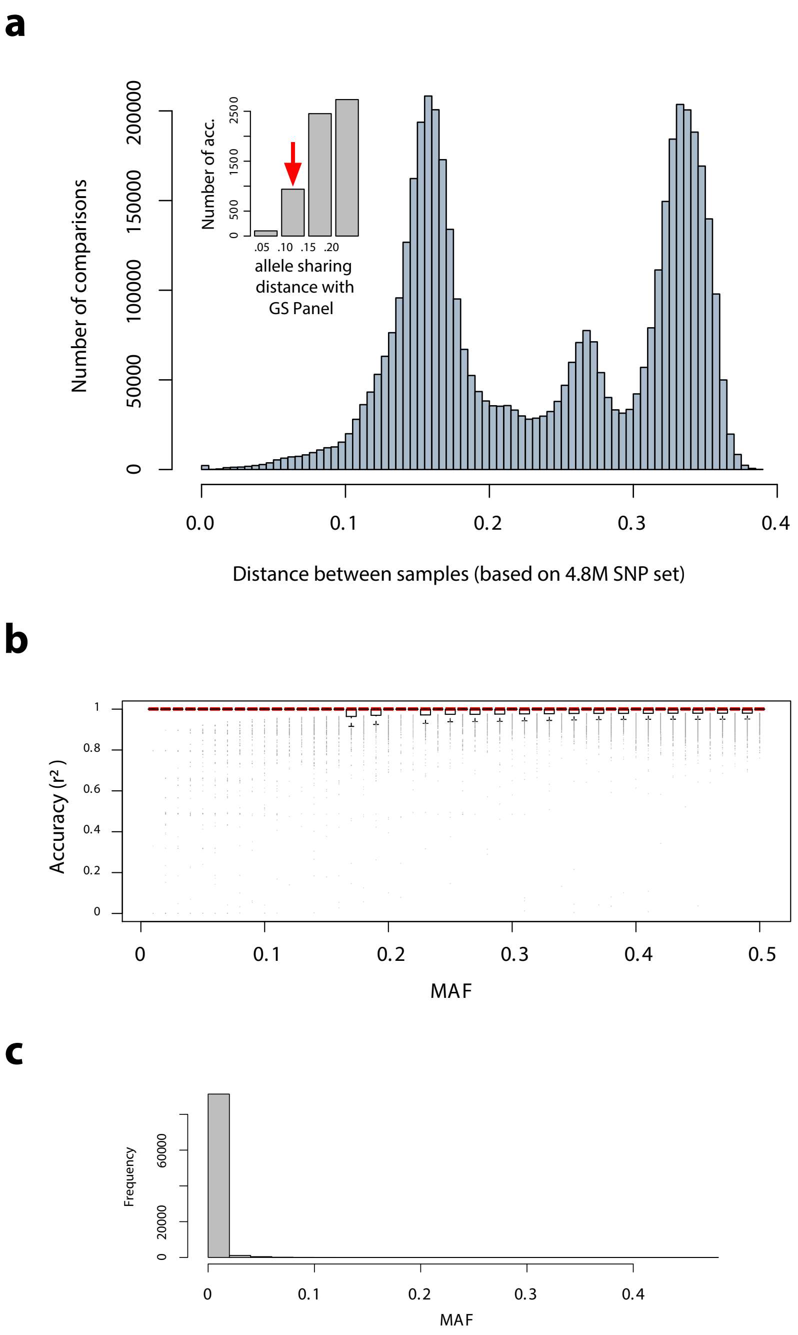

Supplementary Figure 3. Effect of excluding potentially related individuals on Gold Standard Panel imputation. Because of the potential of the 3KRG Panel harboring individuals related to the Gold Standard Panel even after removal of biological replicates, imputation was repeated after removal of 939 individuals with <10% distance with Gold Standard individuals. **(a)** Distribution of pairwise distance in the 3KRG, showing a trimodal distribution consistent with the subpopulation structure of rice: the leftmost peak represents within-Varietal Group comparisons, the rightmost peak represents *Indica-Japonica* cross comparisons, and the middle peak represents comparisons that include admixed individuals. Inset panel indicates the total number of unique accessions in the 3KRG that are potentially related to the Gold Standard Panels at different distance thresholds. **(b)** Imputation accuracy by minor allele frequency of the Gold Standard Panel after removal of 939 potentially related individuals in the reference panel (indicated by red arrow in (a). **(c)** Because r2 cannot be assessed for markers imputed as monomorphic due to lack of variance, 'true' minor allele frequency from the original dataset was examined for these markers. Nearly all markers were truly monomorphic while a total of 4505 polymorphic sites were incorrectly imputed as monomorphic.

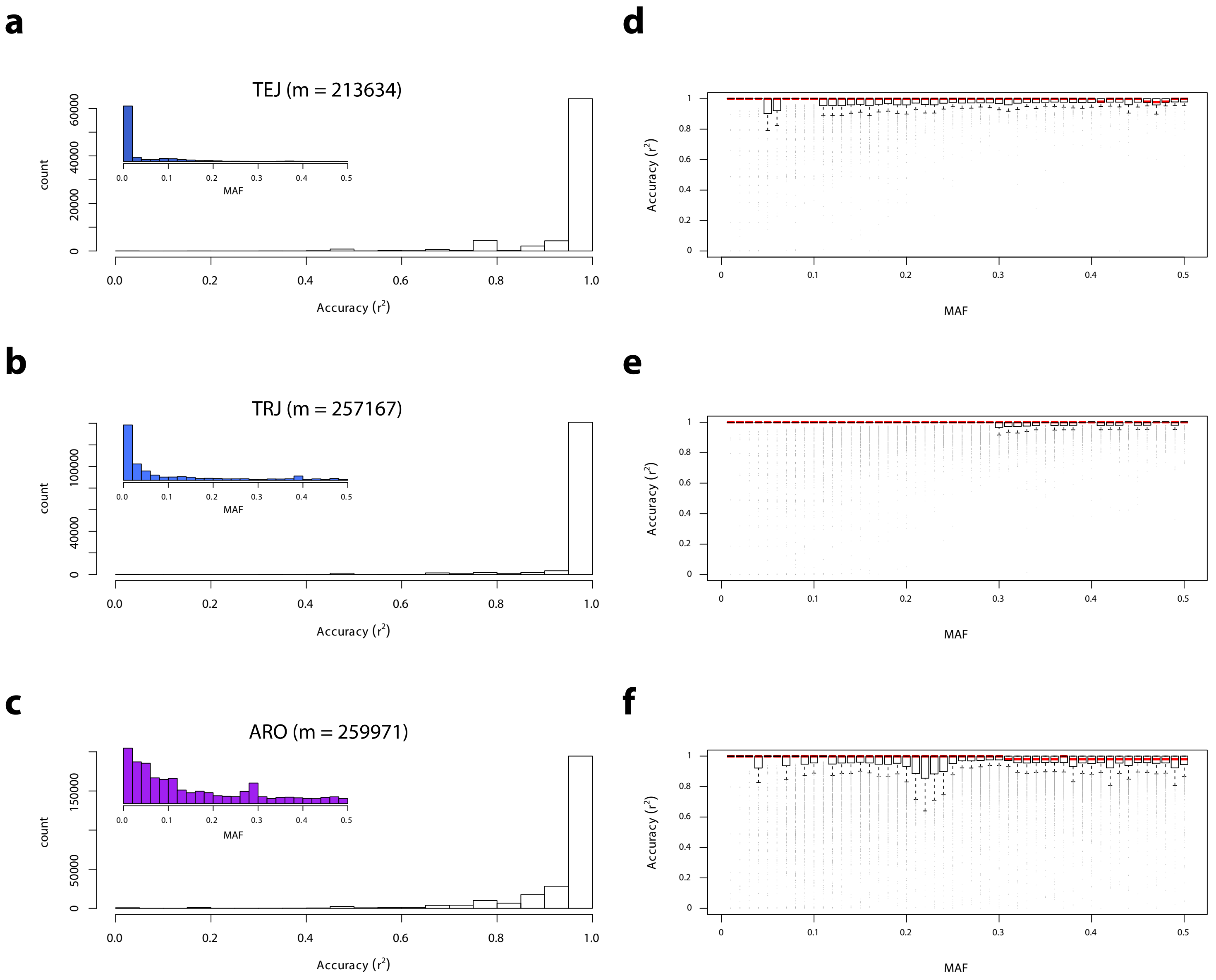

Supplementary Figure 4. Subpopulation-specific imputation accuracy within the Japonica Varietal Group for chromosome three. Overall accuracy (site-based squared correlation) distribution depicted with uncolored histogram plots **(a-c)**. Colored inset histograms display the distribution of minor allele frequency of the within-subpopulation polymorphic markers determined from the 3KRG Panel (total count indicated within parentheses plot title). Boxplots **(d-f)** plot the accuracy distributions across the minor allele frequency (MAF) spectrum in the 3KRG panel (bin size = 0.01; red line = median accuracy value).

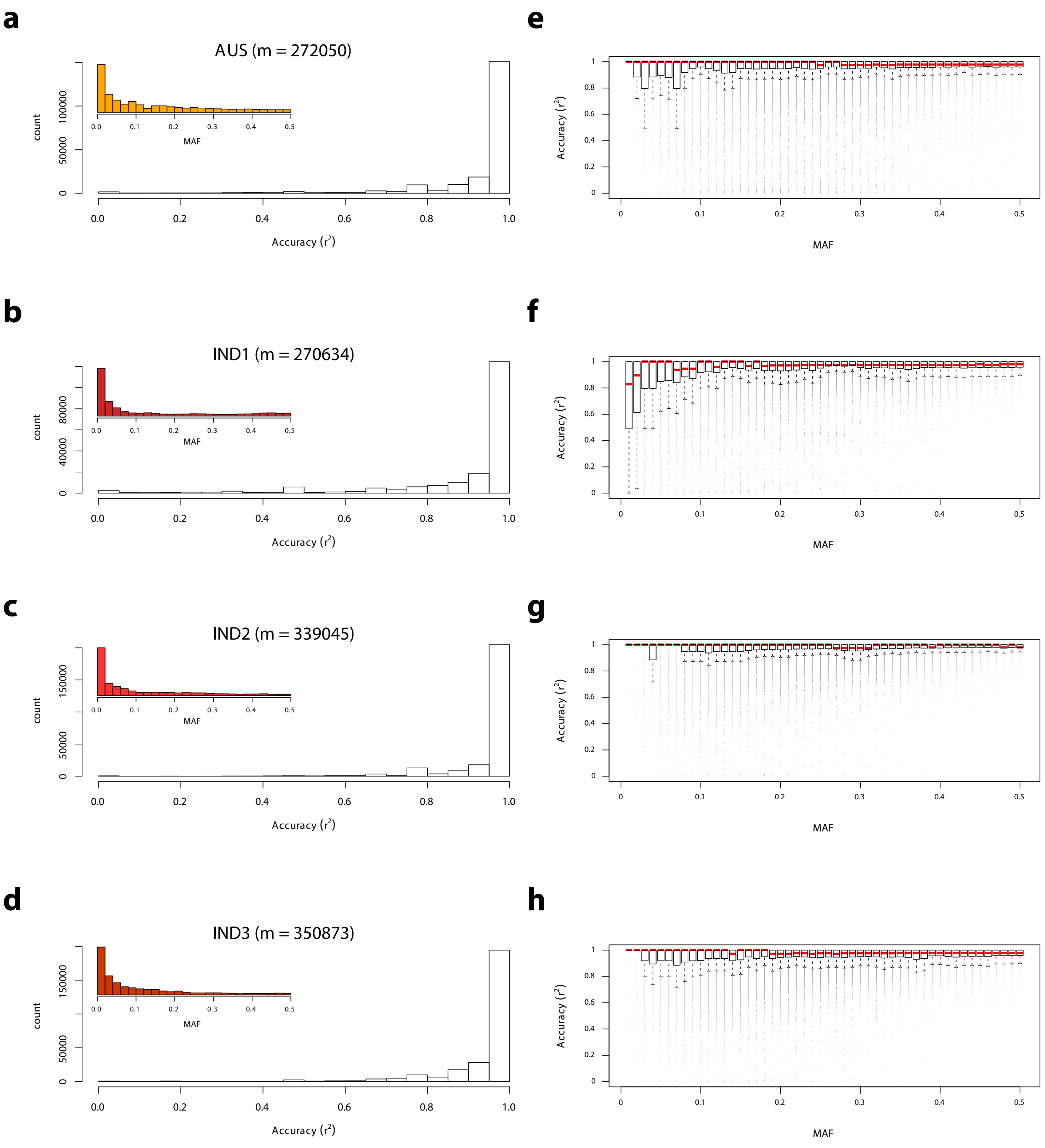

Supplementary Figure 5. Subpopulation-specific imputation accuracy within the *Indica* Varietal Group for chromosome three. Overall accuracy (site-based squared correlation) distribution depicted with uncolored histogram plots **(a-d).** Colored inset histograms display the distribution of minor allele frequency of the within-subpopulation polymorphic markers determined from the 3KRG Panel (total count indicated within parentheses plot title). Boxplots **(d-h)** plot the accuracy distributions across the minor allele frequency (MAF) spectrum in each subpopulation from 3KG panel (bin size = 0.01; red line = median accuracy value).

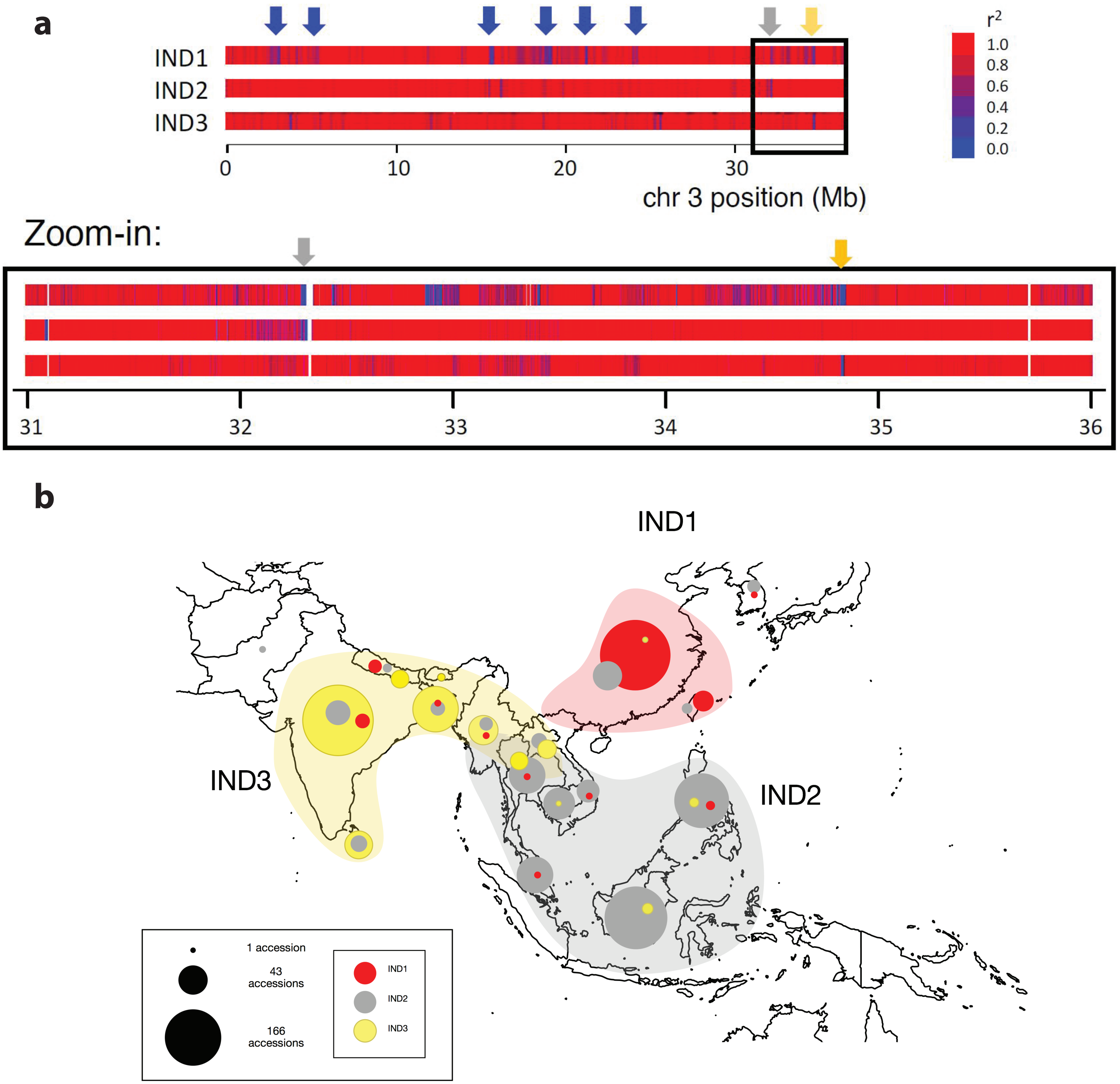

Supplementary Figure 6. Comparison of IND1, IND2, and IND3 *indica* subgroups. The smallest *indica* subgroup, IND1, had lower imputation accuracies of chromosome three at markers of low minor allele frequence compared to accuracies of IND2 and IND3 (Supplementary Fig. 5b-d). **(a)** Examination of imputation accuracy (site squared correlation, r^2) in relation to physical position on chromosome 3. IND1 appears to have about half a dozen regions of low imputation accuracy (arrows), one of which is shared with IND2 (gray arrow) and one of which is shared with IND3 (yellow arrow). Zoom-in plot depicts region 31-36Mb. **(b)** Geographical association of the three *indica* subpopulations in Asia. IND1 is localized primarily to China, whereas IND2 and IND3 are more widely distributed across Southeast Asia and South Asia, respectively. Both IND2 and IND3 accessions were additionally found outside of Asia (not shown on map), whereas members of IND1 were restricted to Asia.

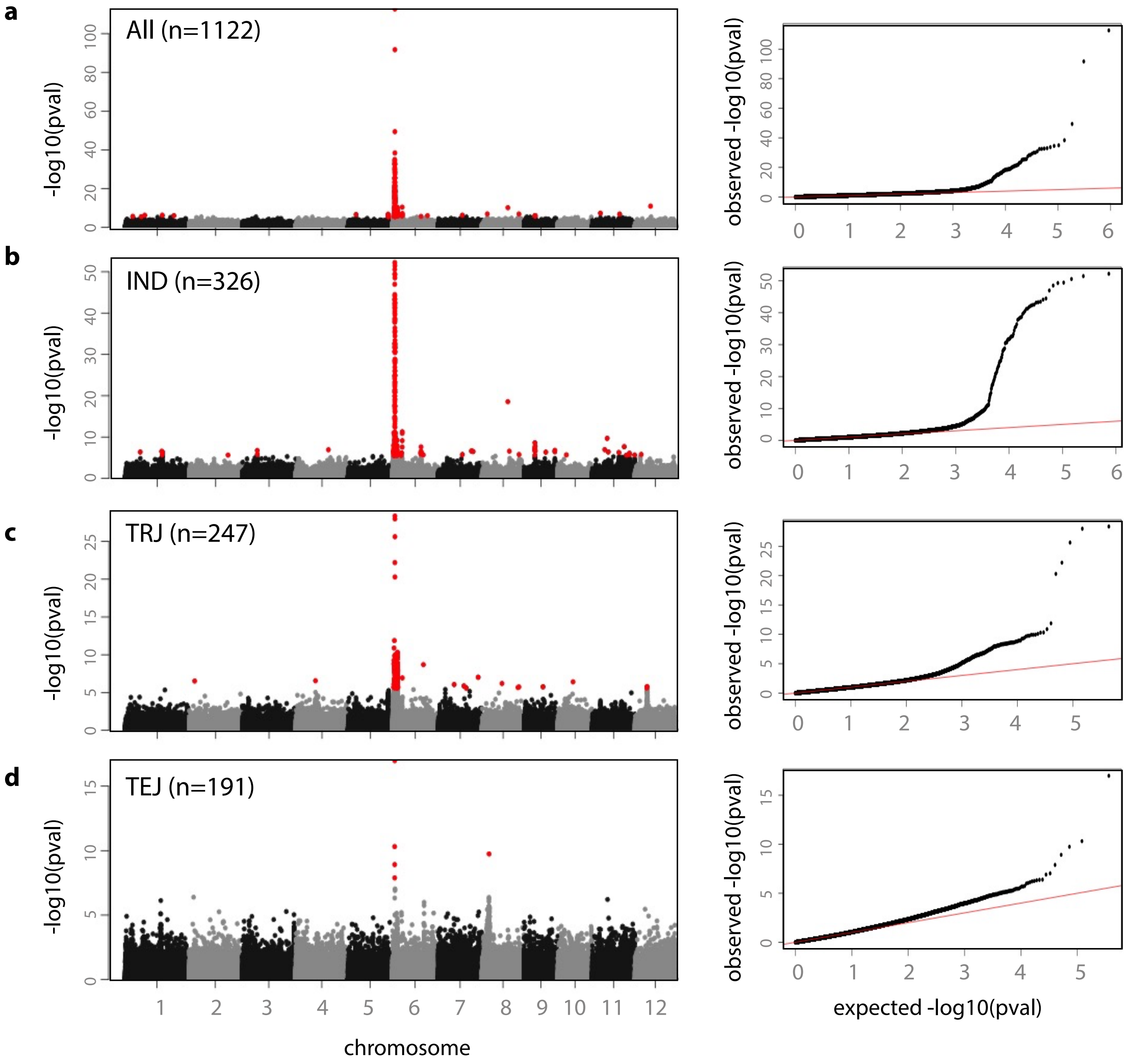

Supplementary Figure 7. Association analysis results for amylose content using un-imputed HDRA data. Left column depicts manhattan plots and right column shows QQ-plots for ALL **(a)**, IND **(b)**, TRJ **(c)**, and TEJ **(d)**. Red SNPs= significant at 1% FDR. Further association analysis was carried out using imputed data on the IND panel (Fig. 2).

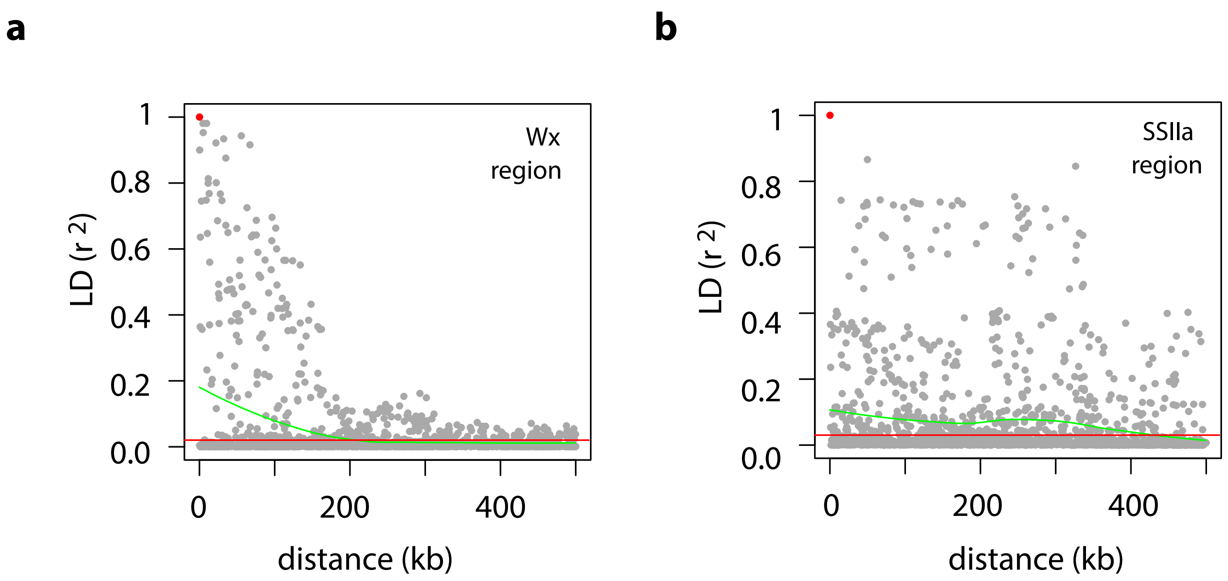

Supplementary Figure 8. Local LD for msSNPs at major and minor association peaks for amylose content. Analysis carried out for the IND panel using original HDRA data for regions containing *Wx* **(a)** and *SSIIa* **(b)**. Green line = 2nd degree Loess curve; red line = SNP-specific critical r2. See Methods for determination of critical r2.

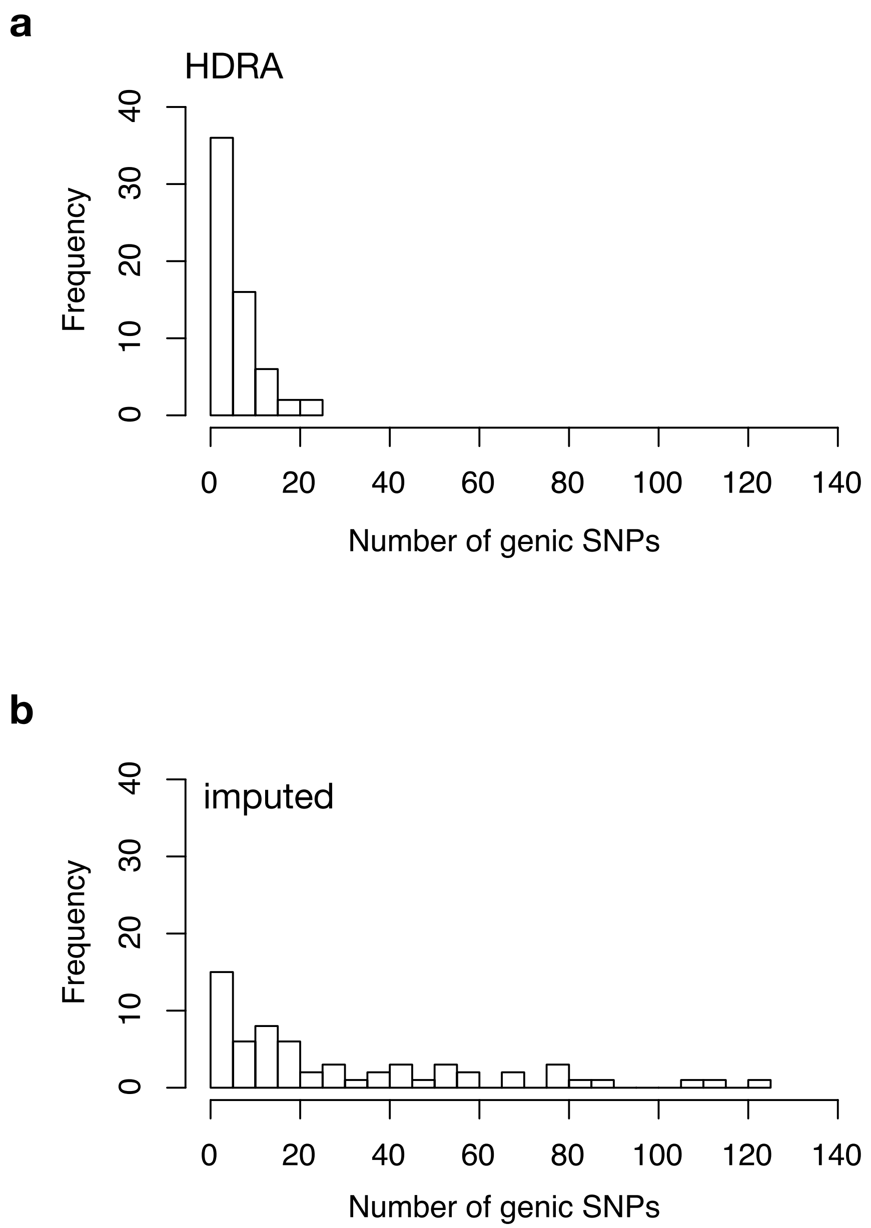

Supplementary Figure 9. Distribution of genic SNPs within gene models underlying the *Wx* association peak. The original 700K SNP dataset **(a)** yielded an average of six SNPs per gene in this region compared to 30 SNPs per gene with the imputed SNP dataset (**b**). Within Wx itself, there were 45 SNPs in the imputed dataset versus 10 SNPs with the unimputed data (**Fig. 4**).

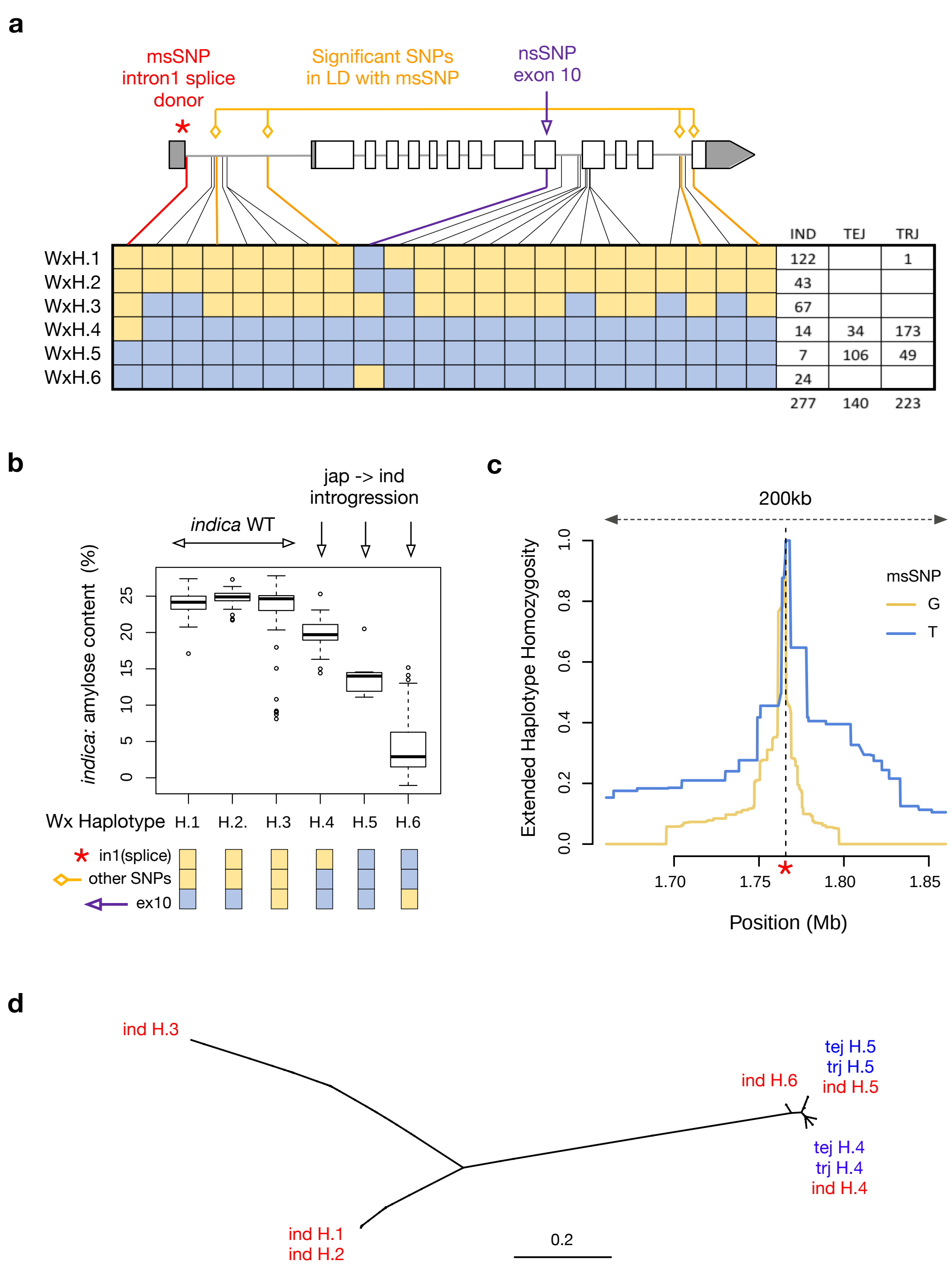

Supplementary Figure 10. Gene haplotype analysis of major-effect gene, *Wx*. (a) MSUv7 LOC_OS06g04200.1 gene model (gray box = UTR; white box= translated exon; line = intron). Below the gene model are gene haplotypes of *Wx* in *indica*, *temperate japonica*, and *tropical japonica* subpopulations using the 22 significant SNPs for amylose content discovered from association analysis in *indica* (**Fig. 4**). The top five most significant SNPs found within Wx gene are annotated with red and orange lines (red line and asterisk = msSNP; orange line and diamond = other top SNPs). Purple line and arrow = another previously identified non-synonymous SNP in exon 10.**(b)** Amylose content distributions across the six gene haplotype groups in *indica*. **(c)** Allele-specific Extended Haplotype Homozygosity analysis using the msSNP at position 1765761bp. The analysis covers a 200kb region containing Wx. **(d)** Distance tree generated using the 45 SNPs in *Wx*. (ind = indica, tej = temperate japonica, trj = tropical japonica). The *Indica* H.4-H.6 clearly cluster with *Japonica* accessions that harbor H.4 and H.5.

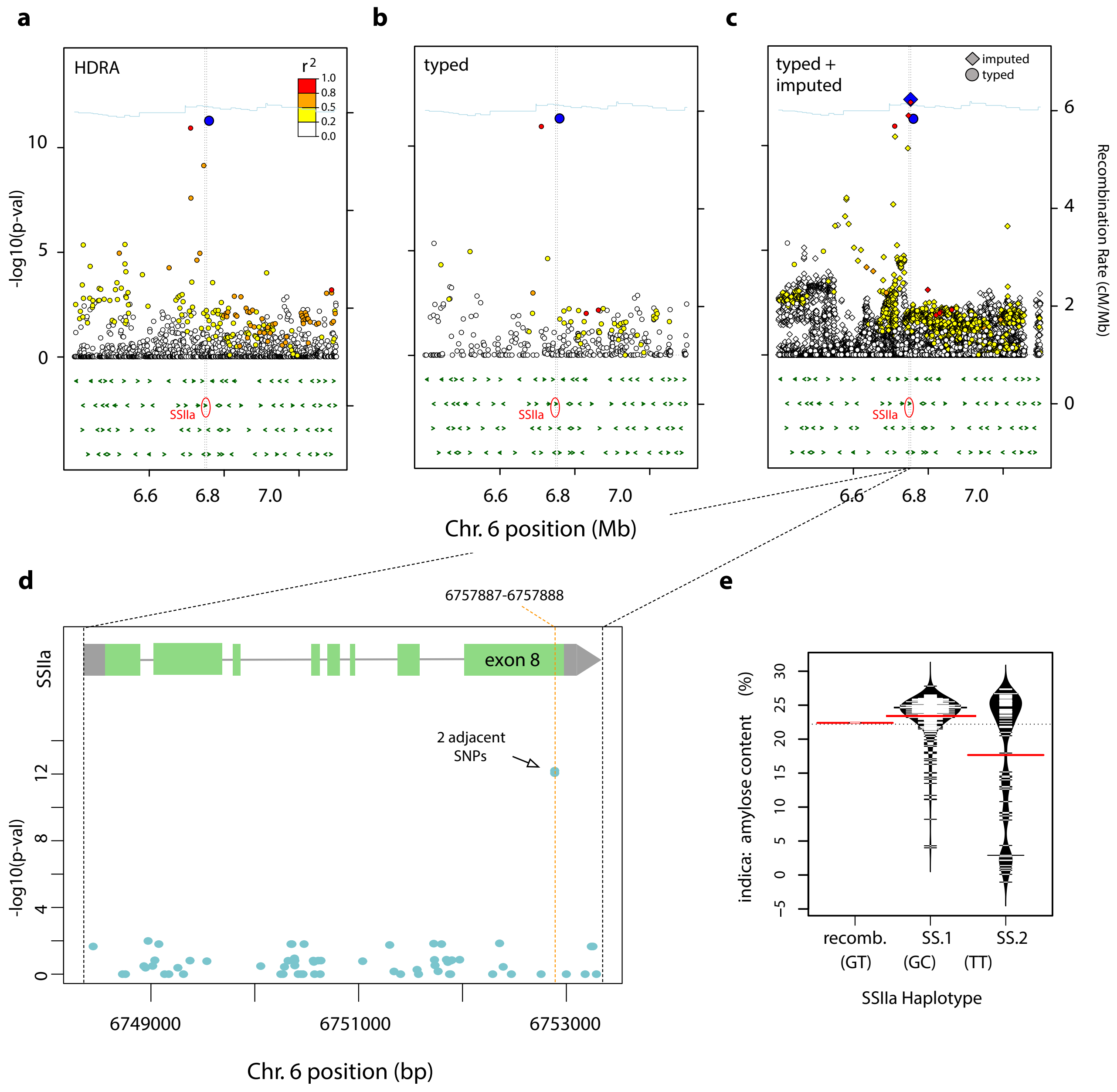

Supplemental Figure 11. Imputed data detects FNP at minor-effect gene, *SSIIa*. (a-c) 700kb regional plots around the SSIIa locus associated with amylose content. Results shown for **(a)** original HDRA data; **(b)** association analysis using imputed data with only typed SNPs displayed; and **(c)** association analysis using imputed data with both typed and imputed SNPs displayed. Colors represent LD (r2) with the msSNPs in the un-imputed and imputed analyses, found at positions 6760419 and 6752887, respectively. Diamonds represent imputed markers while circles represent typed markers. The blue diamond in (c) represents the msSNP for the imputed association analysis. This msSNP, along with adjacent significant SNP at 6752888 matches a previously reported functional GC/TT polymorphism. **(d)** Zoom-in view of association analysis results at the SSIIa gene. Two significant SNPs localized in exon 8 are annotated with the dashed orange line. **(e)** Beanplot showing the phenotypic distribution of SSIIa haplotype classes at the FNP. Red line = mean of each group. Only one individual showed the recombinant haplotype so there is no distribution in the first class.

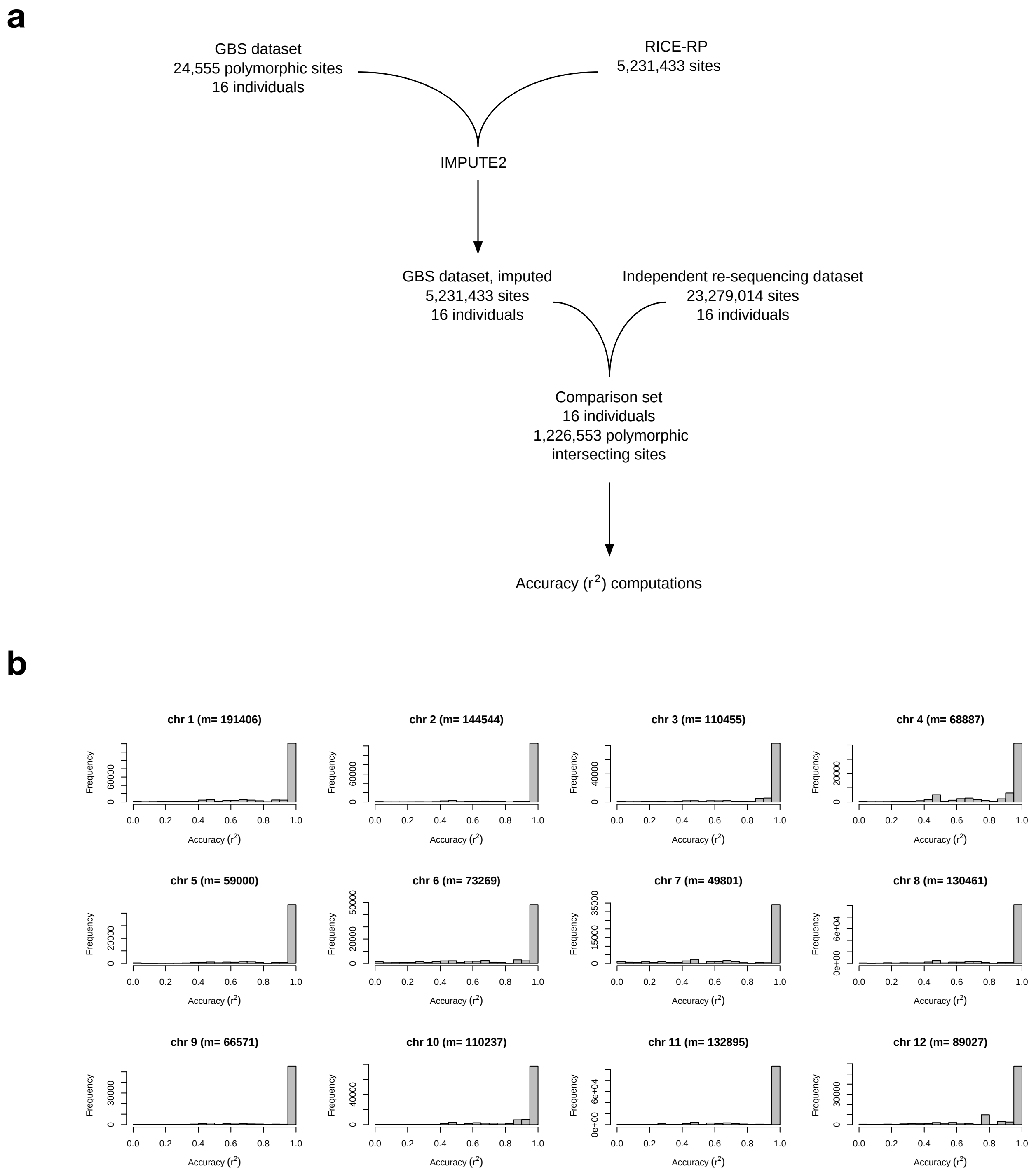

Supplementary Figure 12. Imputation of a Genotyping-By-Sequencing dataset using the Rice Reference Panel (RICE-RP). (a) Overview of experiment. Genomic data from a Genotyping-By-Sequencing project on 16 *tropical japonica* individuals were imputed out to 5.2M SNPs using RICE-RP as a reference panel. Resulting imputed dataset were intersected with an independent, previously published re-sequencing dataset and 1,226,553 intersecting markers polymorphic in both datasets were used for r^2 computation. **(b)** Distribution of imputation r^2 across the 12 chromosomes.

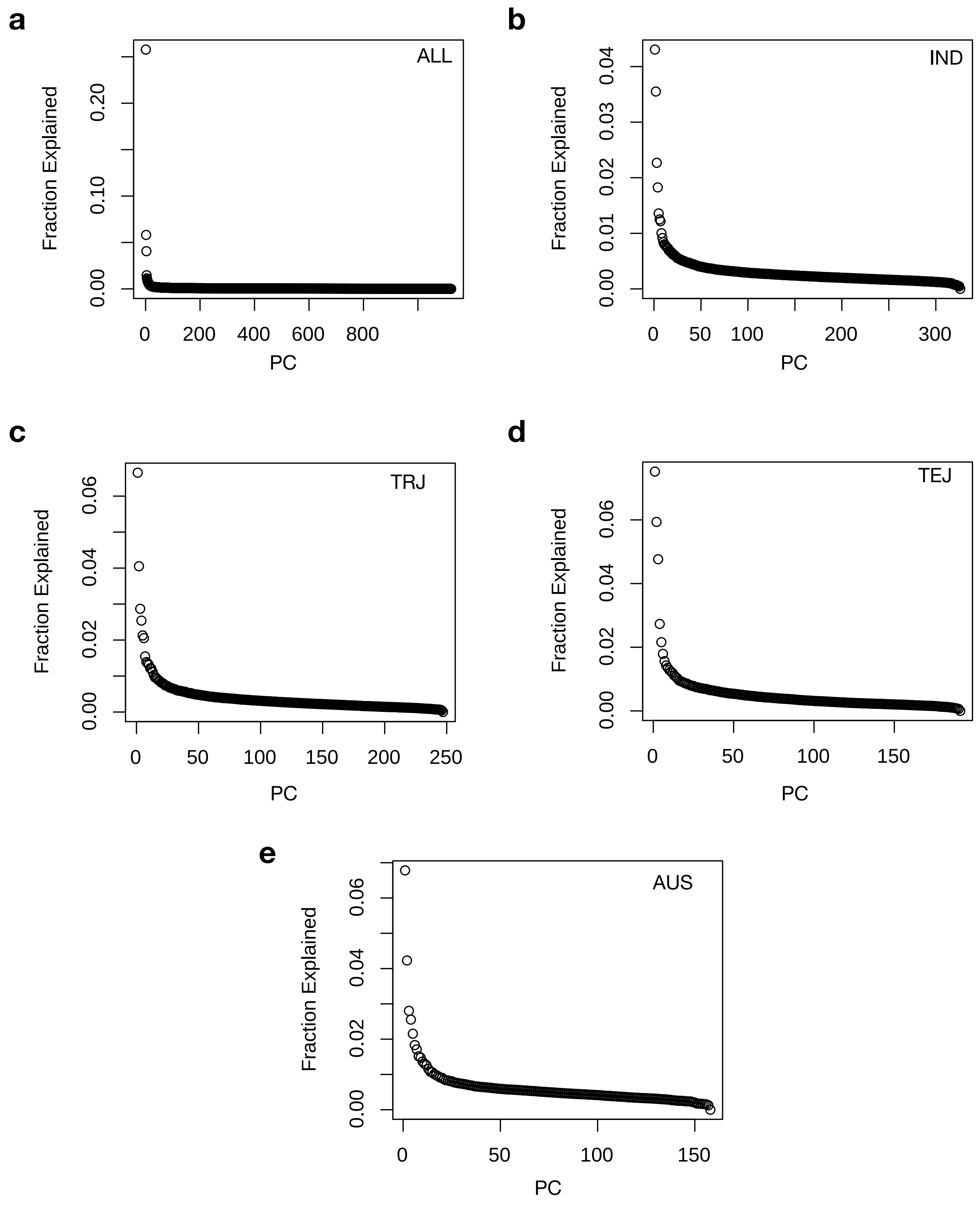

Supplementary Figure 13. (a-e) Scree plots used for determining the number of components to include in GWAS models to control for high-level population structure. Y-axis depicts the proportion of total variance explained by each PC.

Supplementary Table 1. Accessions in the Gold Standard Panel used for optimizing imputation parameters of IMPUTE2. "common_desg" refers to the accession name; "HDRA_subpop" refers to the subpopulation identifier used in the original HDRA Panel; "pair_id" indicates sample identifiers used in the 3KRG and HDRA Panels separated with '@'.

common_desg	HDRA_subpop	pair_id
IR 47686-09-01-B-1	tropical-japonica	IRIS_313-8003@af014bc6.0
IRAT 234	tropical-japonica	IRIS_313-7922@79f5efca.0
VARY MADINIKA 3566	tropical-japonica	IRIS_313-7994@29996530.0
IRAT 112	tropical-japonica	IRIS_313-7914@62fc3268.0
IR 71525-19-1-1	tropical-japonica	IRIS_313-7909@7b0d63f5.0
IR 71524-44-1-1	tropical-japonica	IRIS_313-8010@2793c2ae.0
IR 63380-16	tropical-japonica	IRIS_313-7902@c99e3bb1.0
VIETNAM 1	admixed-japonica	IRIS_313-8011@44046f80.0
VARY LAVA 90	tropical-japonica	IRIS_313-7992@3f191e83.0
VARY LAVA DE BETAFO	tropical-japonica	IRIS_313-7993@05a70989.0
AMARELO	temperate-japonica	IRIS_313-9814@c840a160.0
MITSANGANAHIJERY	tropical-japonica	IRIS_313-7959@dd3201c5.0
IRAT 104	tropical-japonica	IRIS_313-7912@2783f020.0
IR 68704-145-1-1-B	tropical-japonica	IRIS_313-7907@6df8173f.0
IRAT 335	tropical-japonica	IRIS_313-7924@264ca5bc.0
KAKANI 2	admixed	IRIS_313-7933@96d3e4cd.0
MADINIKA 1329	indica	IRIS_313-7725@69c2299e.0
IR 72967-12-2-3	indica	IRIS_313-7911@1259ff35.0
GAMBIAKA	indica	IRIS_313-7620@edd916ea.0
CUIABANA	tropical-japonica	IRIS_313-7870@6e49e6e7.0
VARY LAVA DE MAROVATO	admixed-indica	IRIS_313-7799@658e838e.0
GEANT W 7	admixed	IRIS_313-9817@423be591.0
H 15-23-DA	indica	IRIS_313-7681@4f15d8d7.0
DANAU LAUT TAWAR	indica	IRIS_313-7668@0634f5e4.0
SEBERANG MR 77	indica	IRIS_313-7773@a1260394.0
UPL RI 7	indica	IRIS_313-7797@8ae7984c.0
BODA 148-3	admixed-japonica	IRIS_313-7850@35b48cd4.0
IR 1561-228-3-3	indica	IRIS_313-7684@435c63e3.0
TSIPALA MENA 626	indica	IRIS_313-7795@7743e15d.0
TSIPALA B 160	indica	IRIS_313-7793@b8626bf8.0
WAS 170-B-B-1-1	indica	IRIS_313-7807@34af0d02.0
IR 57924-24	indica	IRIS_313-7699@6c6075ef.0
WAS 198-B-3-1-3	indica	IRIS_313-7815@9546bb86.0
WAS 199-B-1-2-1	indica	IRIS_313-7816@da672708.0
IR 19746-28-2-2	indica	IRIS_313-7685@f4ced436.0
SONA	indica	IRIS_313-7780@0c1db998.0
GOGO	tropical-japonica	IRIS_313-7885@2257c553.0
SALUMPIKIT	indica	IRIS_313-7769@31686493.0
WAS 33-B-B-15-1-4-5	indica	IRIS_313-7826@ab8d68e9.0
KOGONI 91-1	indica	IRIS_313-7719@1b9287de.0
ROJOKELY	admixed-indica	IRIS_313-7758@4bd6d76f.0
MAMORIAKA 114	indica	IRIS_313-7728@cbe9c062.0
CT 6510-24-1-2	indica	IRIS_313-7665@c979ccb5.0
CICA 8	indica	IRIS_313-7664@fd0165e0.0
SAMBALA MALO	indica	IRIS_313-7770@441fd56b.0
WAS 173-B-B-6-2-2	indica	IRIS_313-7808@c4aeea84.0
IR 57920-AC 25-2-B	indica	IRIS_313-7698@435bedf1.0
CHA LOY OE	admixed-japonica	IRIS_313-7856@d6a5eaee.0
IR 55411-50	indica	IRIS_313-7696@ebb77186.0
WAS 174-B-3-5	indica	IRIS_313-7809@7a6814b3.0

Supplementary Table 2. Imputation accuracy of chromosomes 1, 3, and 12. Gold Standard Panel imputation results using selected parameter settings of Ne = 10000, k=100 and a reference panel of size 2973. Distributions are for site-based r2 between imputed calls on the Gold Standard Panel HDRA individuals and true calls on their 50 biological replicates genotyped on the 3000 Rice Genomes Project.

Chr	0th quantile	25th quantile	50th quantile	75th quantile	100th quantile
1	1.60E-04	1	1	1	1
3	2.14E-40	1	1	1	1
12	4.20E-04	1	1	1	1

Supplementary Table 3. msSNPs and their local LD for the two main association peaks for amylose content determined through GWAS with HDRA data

locus	msSNP	pos	baseline critical value r2	LD	start	end
wx	SNP-6.1668314.	1669314	0.02019282	207190	1462124	1876504
SSIIa	SNP-6.6759419.	6760419	0.03020064	431180	6329239	7191599
unknown	SNP-6.3310245.	3311245	0.0145052	62370	3248875	3373615
