# Supplementary material for: An imputation platform to enhance integration of rice genetic resources
Source: Nat Commun. 2018 Aug 29;9:3519. doi: 10.1038/s41467-018-05538-1 (PMC6115364; doi:10.1038/s41467-018-05538-1)
Supplement: Supplementary file 3 — Description of Additional Supplementary Files [file 41467_2018_5538_MOESM3_ESM.pdf]

Descriptions of Additional Supplementary Information

File Name: Supplementary Dataset 1
Description: HDRA and 3KG Germplasm Collections ("RDP1," "RDP2, "3KG"). 4591 O. sativa samples.

File Name: Supplementary Dataset 2
Description: Concordance tables from HDRA Panel imputation. Each chromosome was imputed in 15 equally-sized chunks.

File Name: Supplementary Dataset 3
Description: Gene models found within the region of local LD to the msSNPs at the major and minor association peaks for amylose content using original HDRA data.

File Name: Supplementary Dataset 4
Description: Wx gene haplotypes in IND, TEJ, and TRJ
